# Acute exposure of 532 nm laser differentially regulates skin tissue transcription factors

**DOI:** 10.1371/journal.pone.0230175

**Published:** 2020-03-19

**Authors:** Rajkumar Tulsawani, Purva Sharma, Niroj Kumar Sethy, Pooja Kumari, Lilly Ganju, Satya Prakash, Satish Chouhan

**Affiliations:** 1 Defence Institute of Physiology and Allied Sciences, Delhi, India; 2 Laser Science and Technology Centre, Metcalfe House, Delhi, India; Massachusetts General Hospital, UNITED STATES

## Abstract

High energy laser, particularly 532 nm, is widely used in defense and medical applications and there is need to address its occupational safety. Thermal and non-thermal effects of 532 nm high energy laser on skin are cause of concern. This study indicates impact of 532 nm laser on rat skin and first of its kind of attempt to understand transcriptional activation of genes as an early response following laser exposure. Skin of experimental rats were exposed to 532 nm radiance at 0.1, 0.25 and 0.50 W/cm^2^ for 10 sec. Thermographic changes of skin exposed to 532 nm laser exhibited increased Tmax temperature in radiance dependent manner. After thermal imaging, skin of experimental rats was collected 1 h post laser exposure for studying differential gene expression. The skin exposed to lower power density (0.1 W/cm^2^) did not show significant changes in expression of gene pathways studied. At moderate radiance (0.25 W/cm^2^), predominantly canonical wnt/B-catenin pathway genes notch1, axin2, ccdn1, wnt5a and redox homeostasis genes; txn1, nqo1 and txnrd1 were expressed. At higher radiance (0.5 W/cm^2^), significant repression of genes related to wound healing process particularly notch/wnt pathway viz. hes5, wnt1, wn3b with higher expression of dab2 was recorded. The data obtained from these studies would help in drawing safety limits for skin exposure to 532 nm laser. Further, genes expressed at moderate and high level of radiance exposure to skin were distinct and differential and provide new avenue to configure pathway to counteract laser induced delay in tissue injury and hair follicular damage.

## Introduction

Expansion in the array of laser technology requires safety studies based on the biological experimental data to contribute towards laser safety standards [1]. High energy lasers including 532 nm has defence, industrial and medical applications [2–4]. The damage to biological tissue by high power laser depends upon the wavelength, power density, length of exposure and depth of penetration [5]. Lasers operating in the visible spectral region particularly 532 nm has been widely used in medical technology such as skin rejuvenation, platelet aggregation, laryngeal lesions, acne treatment, tattoo and pigmentation control, prostate surgery, cardiovascular surgery, vascular lesions, glaucoma treatment, tissue remodeling, reduction in facial wrinkles etc. [6–20]. Nevertheless, biological effects of laser signify it as a competent tool as well as a potentially hazardous device presenting with unforeseen occupational risks to operators and ancillary personnel [21]. In general, impact of laser on eye is considered significant than skin, however, exposure of high energy laser such as 532 nm to skin may lead into burn injuries. Further, being large exposure area of skin in body of organism, probability of exposure of laser is much more on skin than precise focusing on retina. Thermal damage to irradiated skin is primarily due to local absorption of laser energy in skin tissue leading to denaturation of tissue and may cause cellular malfunctions [22,23]. Laser irradiation leads to heat generation in tissue upon absorption [24]. Laser induced heat may cause reversible and irreversible damage in biological tissue. The low heat transfer by laser exposure activate cellular defence mechanism and are mostly reversible in nature however severe heat exposure may lead to immediate (primary) or after (secondary effects) [24,25]. Additionally, low level of laser exposure may lead to apoptosis, signal transduction and activation, however, high power laser cause necrotic death [24,25–27]. Heat exposure causes protein degradation and DNA damage in cells, leading to genetic modifications and cell death, also loss of viability has been reported in the surrounding tissue owing to heat diffusion [28].

Numerous studies have been carried on the impact of near-infrared laser on skin of experimental animals [2,22,23,29–35] but the studies on impact of 532 nm on skin tissue are sparse. Therefore, the present study has been designed to address the data gap on 532 nm laser at morphometric and genetic level using rat skin model.

## Materials and methods

### Animals

All experimental procedures conducted on rats were approved by Institutional Animal Ethical Committee (DIPAS/IAEC/2015/07), DRDO, Delhi, India. Male Sprague-Dawley rats weighing 150–180 g were obtained from Institutional Animal Facility. Three rats per group were used for gene expression studies and six rats per group were used for thermography, biochemical and protein expression studies. They were fed on standard chow and water *ad libitum* and were maintained under controlled environment with constant temperature 25 ± 1°C and humidity 55% ± 10% and a 12 h light/12 h dark cycle at the Institute’s animal house. All experiments were performed in accordance with relevant guidelines and regulations.

### Experimental animals and laser irradiation

Experimental animals were anesthetized using ketamine-xylazine cocktail and dorsal portion of rat skin was shaved with electric razor to avoid tissue hair interaction with laser exposure. The shaved dorsal skin was divided into four grids for laser exposure using marker pen. In each experimental animal, four skin grids were exposed to 532 nm CW laser ND:YAG laser at various power densities (unexposed, 0.1 W/cm^2^, 0.25 W/cm^2^ and 0.5 W/cm^2^) for 10 sec exposing 10 mm diameter spot from 10 cm distance. The experimental animals regained consciousness within 5–10 min of exposure. After 1 h of exposure period, 10 mm laser exposed skin tissue of 3–4 mm depth was collected under anesthesia. The incision wound was sterilized with 70% isopropyl alcohol. All rats were euthanized after collection of skin with sodium pentobarbital in booster doses as required. The laser exposure density was selected based on preliminary studies carried out in laboratory in which laser radiance above 1 W/cm^2^ was found to be harmful for the animals.

### Skin temperature measurement

Male Sprague-Dawley rats weighing 150–180 g were anesthetized using ketamine-xylazine cocktail and fur over the dorsal portion of experimental animals were shaved 24 h prior to exposure. Before and after laser exposure, baseline and post exposure local skin temperature was monitored using an infrared video camera (VarioCam, Germany) with spectral range of 7.5–14 micrometer and measures temperature in the range of -40°C to +600°C with 2% accuracy.

### Preparation of tissue homogenates and gene expression studies

Skin tissue homogenate was snap frozen and RNA was isolated using RNA isolation kit (QI Amp RNA Blood Mini Kit, Qiagen, US). RNA was transcribed into cDNA using commercial kit (Qiagen, US, cat. no. 330401 RT2 First Strand Kit). The gene expression studies were performed using Qiagen signal transduction kit (Qiagen US, Cat No. PARN-014ZC). Gene expression of Ppard and Hmox1 was evaluated using TaqMan probes (Rn00565707_m1 and Rn00561387_m1 respectively) with Gapdh (Rn01775763_g1) as endogenous control. The reactions were carried out in a StepOne Plus real time PCR system (Applied Biosystems) using TaqMan universal master mix (Cat No. 4364338, Thermo Fisher) as per manufacturer's recommendations.

### Biochemical and protein expression studies

The total antioxidant status was measured as indicator of stress induced by the 532 nm laser exposure using commercial estimation kit (BioAssay DTAC-100). Quantitative Elisa kits were used for studying expression of heme oxygenase 1 (HO1) and erythropoietin EPO following the manufacturer’s instructions (R&D Systems, USA). Briefly, equal amounts of protein samples were loaded onto each specific antibody coated wells and after incubation period, a horseradish peroxidase-conjugated secondary antibody was added. The reaction was completed with TMB and stop solution and finally, color generated was read at 450 nm against the specific standards provided in the kit.

## Results and discussion

Though it is widely considered that eye particularly retina is the primary target for the high energy laser including 532 nm wavelengths however, damage to skin is equally important owing to its larger exposure area. Skin is largest complex organ in the body and protects body against environmental insults and involved in neurological, immunological and endocrine function [36–38]. Skin is composed of epidermis with keratinocytes, melanocytes, Langerhans cells and dendrite cells and dermis with fibroblast, dermal white adipocytes, vasculature and immune cells [38]. Skin epidermal transcription factors such as AP1, PPAR, Smad2, Foxo1, Wnt/B catenin transcription factors regulate cell proliferation, differentiation, apoptosis and migration [39–44] which further activates wnt/catenin pathway in dermis [43,45]. An insult to skin by strong stimuli or stressors including radiations, lead to activation of cell survival, apoptotic, inflammatory and wound healing pathways. Therefore, the present study was designed to study changes caused by 532 nm laser on skin tissues using rodent test model. Despite, the difference reported in literature about anatomy of rat with humans, rat model is similar to human in term of expression of genes and functionality. Further, the study will fill the data gaps as there is no study on rat conducted so far. In future, the test model can be validated with finding of this study with higher species such as mini pigs having close resemblance with human skin. The use of animal model such as mini pig for experimental purpose is restrictive.

**Fig 1:**
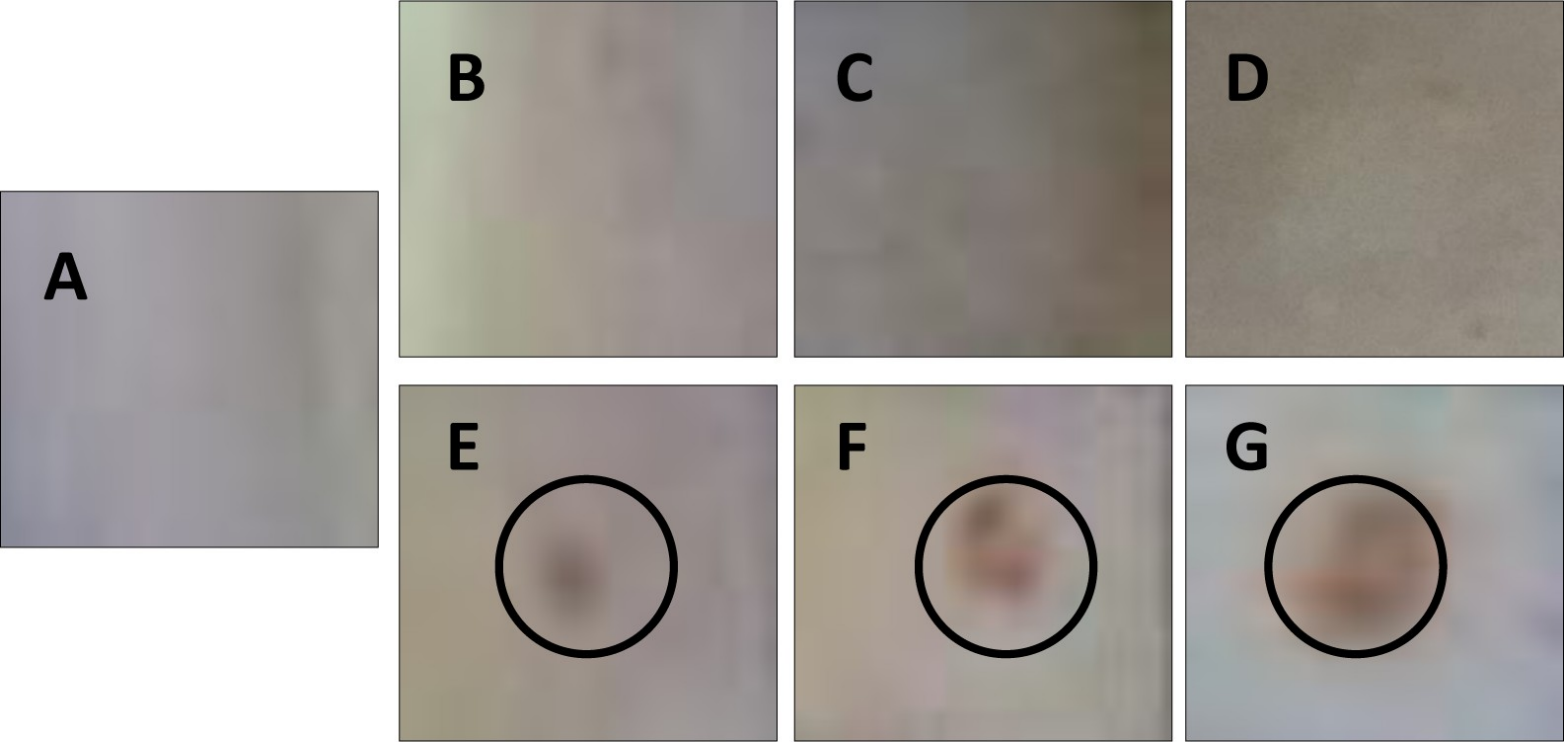
Dosimetry studies of rat skin exposed to 532 nm laser. Fig 1A represent unexposed rat skin while Fig 1B–1F presents shows rat skin exposed to 532 nm laser at various fluences; 0.1 W/cm^2^ (B), 0.25 W/cm^2^ (C), 0.5 W/cm^2^ (D), 1.0 W/cm^2^ (E), 1.5 W/cm^2^ (F) and 2.0 W/cm^2^ (H).

**Fig 2:**
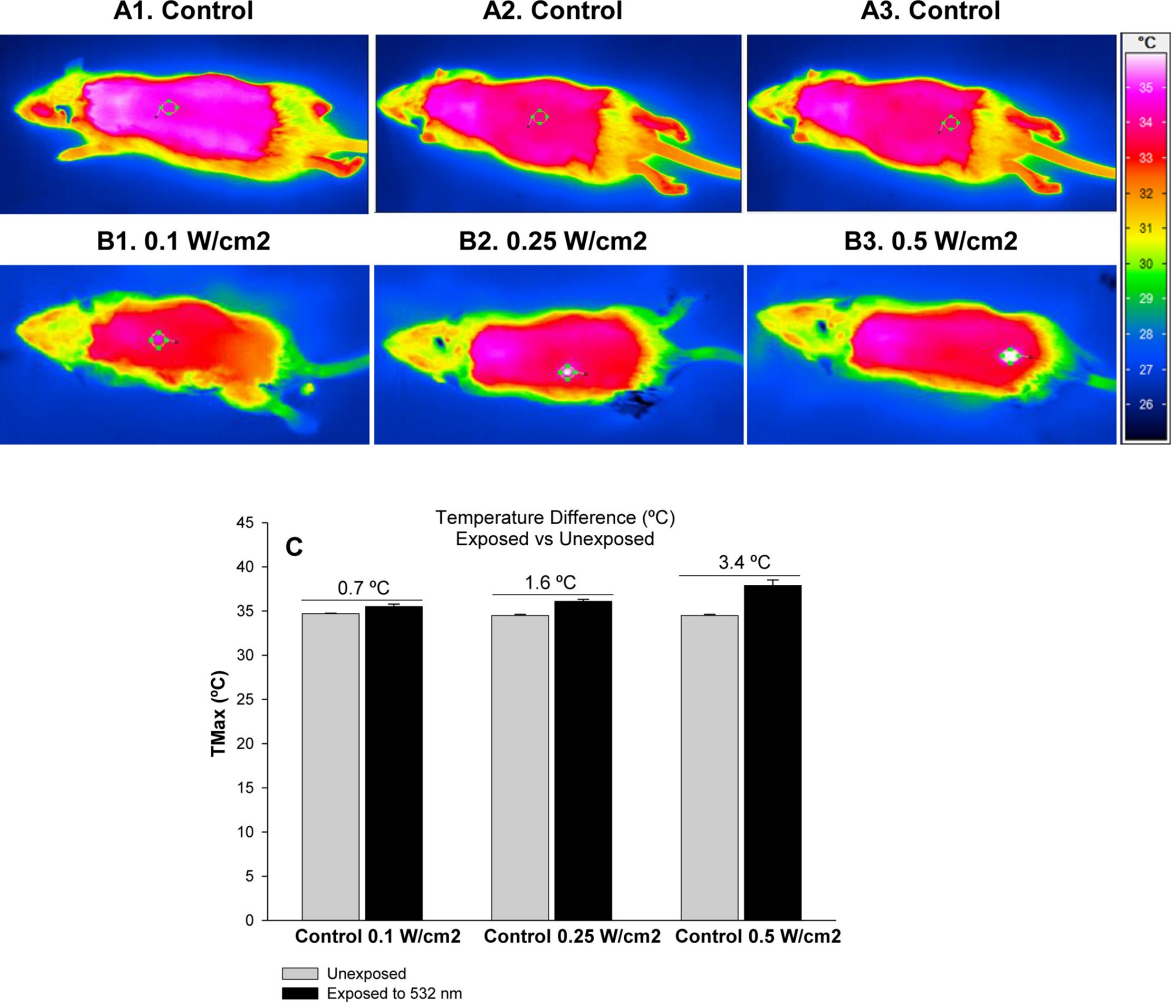
Morphometric analysis and thermal imaging of rat skin exposed to 532 nm laser. Thermogram of exposed rat skin to 532 nm laser at various fluences vs respective baseline thermogram is: 0.1 W/cm^2^ (B1) vs A1, 0.25 W/cm^2^ (B2) vs A2 and 0.5 W/cm^2^ (B3) vs A3. Fig 1C shows Tmax data and temperature difference between exposed and unexposed rat skin.

In preliminary dosimetry studies, rat skin exposer to 532 nm did not reveal visible lesions below 1 W/cm^2^ (Fig 1B and 1C) and were comparable to unexposed rat skin (Fig 1A) however exposure of skin to 532 nm at higher laser power density resulted in redness, burning and ablation in skin at 1 W/cm^2^, 1.5 W/cm^2^ and 2 W/cm^2^ (Fig 1E–1G). Hence, to address safety limits of laser exposures, power densities of 0.1 W/cm^2^, 0.25 W/cm^2^ and 0.50 W/cm^2^ were selected for the studies. Laser exposure induces heat transfer that causes protein degradation and genetic damage [28]. Therefore, thermal imaging was carried out using infrared camera. The rat skin exposed to 532 nm laser at different power densities (0.1–0.5 W/cm^2^) as specified in materials and methods showed radiance dependent inflammatory response particularly at moderate (0.25 W/cm^2^) and high radiance (0.50 W/cm^2^) Fig 2A1–2A3 and 2B1–2B3). The local skin temperature Tmax increased upon increasing radiance exposure from 0.1 W/cm^2^ to 0.5 W/cm^2^. The Tmax temperature was 0.7°C higher compared to unexposed rats which was negligible. However, change in Tmax temperature 1.6°C and 3.4°C at 0.25 W/cm^2^ and 0.50 W/cm^2^, respectively, was notable and raise concern related to exposure to 532 nm high power laser especially at moderate and high radiance (Fig 2C).

Moreover, thermal absorption following high power genetic damage [28] and studies on gene expression post 532 nm exposure was conducted. Wide arrays of genes were selected representing many signal transduction pathways including TGFβ, WNT, NFĸB, JAK/STAT, p53, Notch, Hedgehog, PPAR, oxidative stress and hypoxia signaling. All these pathways are well expressed in skin tissue and associate with skin injury and repair and often cross talk to maintain cell homeostasis. In the present study, experimental rats were exposed to 532 nm radiance at 0.1, 0.25 and 0.50 W/cm^2^ for 10 sec and skin was collected 1 h post laser exposure for studying differential gene expression.

**Fig 3:**
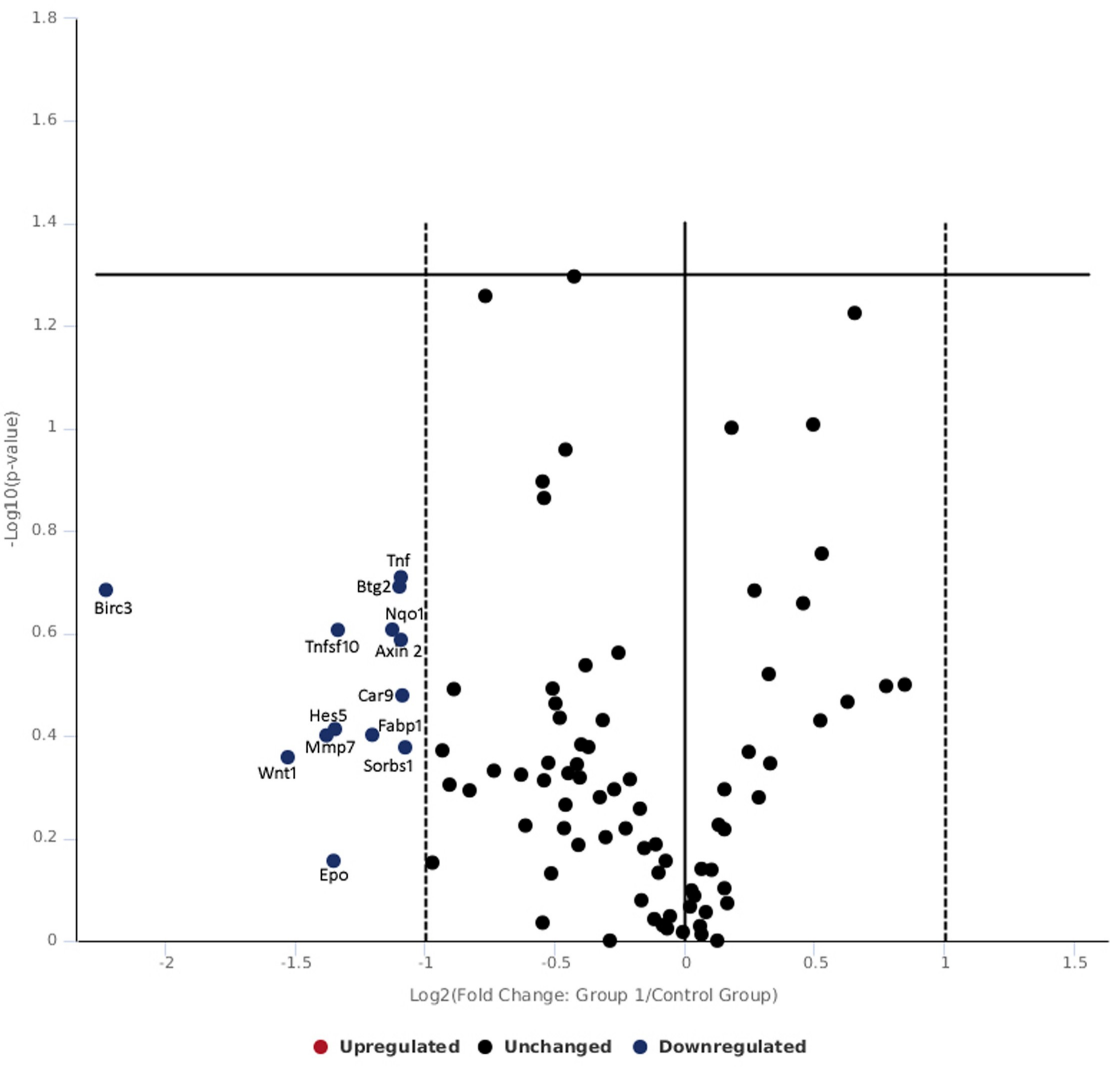
Volcano plot of genes expressed following 532 nm laser exposure at low irradiance of 0.10 W/cm^2^.

Table 1 and Fig 3 shows gene expression of skin tissue exposed to lower radiance 0.1 W/cm^2^. In the present study, the skin exposed to 0.1 W/cm^2^ power density did not show significant changes in expression of gene pathways studied. At this low exposure level of 0.1 W/cm^2^, there was no difference in over expression of genes with respect to control however the following genes were found to be down regulated including hes5 (notch signaling), tnfsf10 (TFG signaling), ifng (NFkB signaling), wnt1 (hedgehog signaling), epo (hypoxia signaling), mmp7 (wnt signaling), fabp1 (PPAR signaling), bbc3 and btg2 (p53 signaling). The fold change in gene expression observed were not appreciable and data finding suggest that there was heat transfer via lower power laser exposure which resulted in configuration and re-configuration in studied genes for negating impact of laser exposure. Further, no genes related to oxidative stress were found to be expressive or under expressed and it can be predicted that fluences of 0.1 W/cm^2^ has not translated in enough heat to cause adverse effect in skin tissue.

**Table 1:** Genes expressed following 532 nm laser exposure at low irradiance of 0.1 W/cm^2^.

Gene Symbol	Fold Regulation	Predictive Pathway
Hes5	- 2.96	notch pathway
Tnfsf1	- 2.43	tfg pathway
Ifng	- 2.30	nfkb pathway
Wnt1	- 2.30	hedgehog pathway
Epo	- 2.30	hypoxia pathway
Mmp7	- 2.30	wnt pathway
Fabp1	- 2.30	ppar pathway
Bbc3	- 2.21	p53 pathway
Btg2	- 2.06	p53 pathway

**Fig 4:**
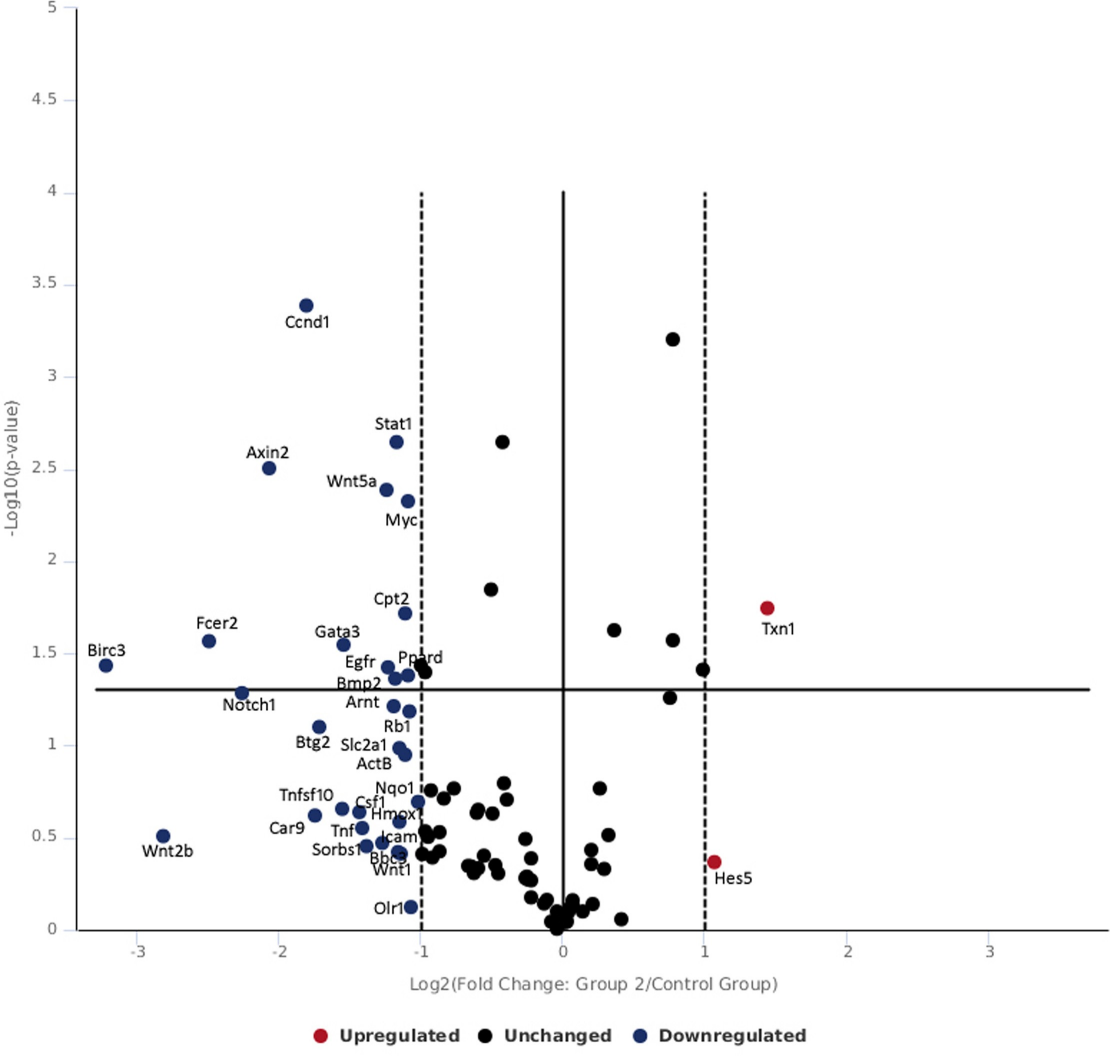
Volcano plot of genes expressed following 532 nm laser exposure at moderate irradiance of 0.25 W/cm^2^.

Table 2 and Fig 4 shows gene expression of skin tissue exposed to moderate radiance 0.25 W/cm^2^. At moderate exposure level at 0.25 W/cm^2^, txn1 was found to be upregulated which was concomitant with the repression of nqo1 and trnxd1. The down regulation of oxidative stress related genes nqo1 and trnxd1 found in this study correlates with the other studies where negative expression of nqo1 and txrnd1 has been found favorable in prognosis in cancer patients [46,47]. The fold change in gene expression observed was not appreciable in gene expression levels suggesting that 0.25 W/cm^2^ induced minor stress but enough to be categorized under severe embarrassment. Wnts play important role in regulation of development and homeostasis via cell autonomous and non-cell autonomous mechanisms [48,49] and act as growth factors to regulate cell proliferation, differentiation, migration and polarity [50–52]. In the canonical wnt pathway, in absence of wnt ligand, β-catenin is degraded via complex of axin, apc and GSK3, p2a, CK1α. Alternatively, level of β-catenin can be stimulated by growth factor such as TGFβ in fibroblast which is released in early phase of wound repair [53]. Canonical wnt signaling involves accumulation and translocation of β-catenin into nucleus [53,54]. In the present study, exposure of 0.25 w/cm^2^ 532 nm laser, caused repression of axin2 which led to β-catenin stabilization and in turn β-catenin repressed its target gene ccnd1 associate with de-differentiation of epithelial cells. Further, notch 1 can inhibit beta-catenin-mediated signaling [55] and wnt5a that regulate inflammatory process in wound repair and works independent of catenin were repressed. The finding suggests that exposure of moderate level of 532 nm laser exposure predominantly canonical wnt pathway that plays role in development, homeostasis and improvement of the wound healing in skin.

**Table 2:** Genes expressed following 532 nm laser exposure at moderate irradiance of 0.25 W/cm^2^.

Gene Symbol	Fold Regulation	p-Value	Predictive Pathway
Txn1	2.50	0.0197	oxidative stress pathway
Hmox1	-2.41		
Nqo1	-2.19		
Txnrd1	-2.10		
Notch1	-3.87		notch pathway
Hey1	-2.15		
Heyl	-2.01		
Axin2	-3.74	0.0134	wnt pathway
Ccnd1	-3.67	0.0039	
Ccnd2	-2.12		
Myc	-2.31	0.0147	
Ppard	-2.31		
Fosl1	-2.02		
Btg2	-3.56	0.0276	p53 pathway
Egfr	-2.54		
Rb1	-2.29	0.0364	
Tnfsf10	-3.18		tgf pathway
Gata3	-3.17		jak/stat pathway
Irf1	-2.06		
Birc3	-2.92	0.0411	nfkb pathway
Icam1	-2.61		
Stat1	-2.45	0.0004	
Csf1	-2.05		
Wnt5a	-2.55	0.0238	hedgehog pathway
Bmp2	-2.45		
Ptch1	-2.01	0.0486	
Arnt	-2.47	0.0310	hypoxia pathway
Slc2a1	-2.40		
Cpt2	-2.34	0.0408	ppar pathway
Sorbs1	-2.25		

**Fig 5:**
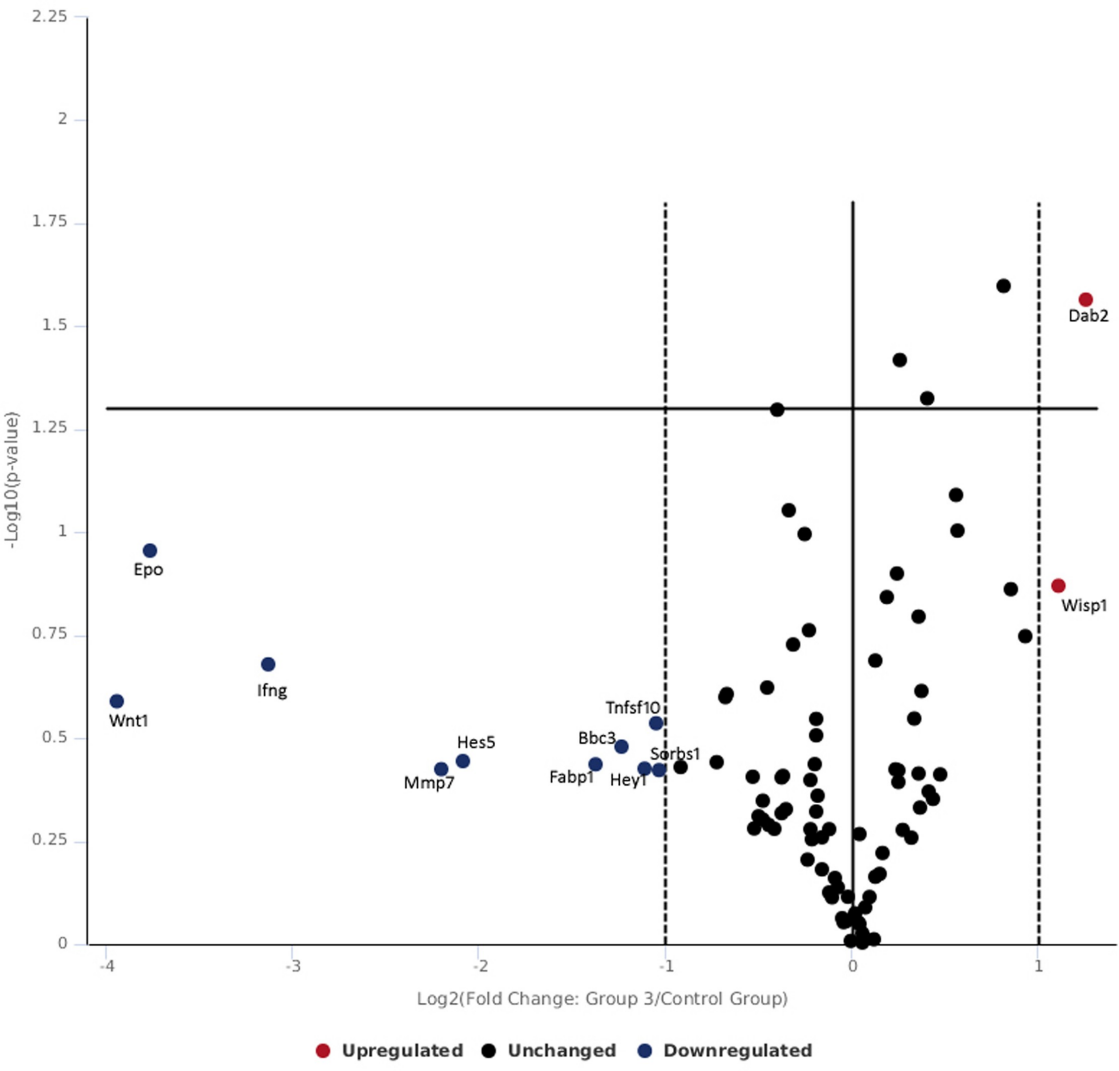
Volcano plot of genes expressed following 532 nm laser exposure at high radiance of 0.5 W/cm^2^.

The skin tissue exposed to 0.5 W/cm^2^ 532 nm laser revealed expression of distinct and differential subset of genes related to wnt signaling which crosstalk with notch, nfkb and hedgehog signaling (Table 3 and Fig 5). It was observed that bbc3 target of p53 pathway was under expressed. p53 has been linked cell growth inhibition and DNA repair and apoptosis in cells / tissue exposed to ionizing radiations and variety of environmental stressor [56]. Cellular DNA damage leads to cell growth arrest in G1 or G2 phases by p53 and provide cells time to repair. In case, cells fail in DNA repair, p53 initiate apoptosis process [57,58]. It has been postulated that expression of p53 has been related with skin ulcers in rat [59]. Hedgehog signaling regulates hair cycling through p53 [60]. In the present study, bbc3 gene which is direct target of p53 has been found to be repressed following exposure of 0.5 W/cm^2^ which evidences the involvement of p53 pathway in skin tissue following 523nm laser exposure (Table 3 and Fig 5). It has been reported that higher bbc3 expression promote apoptosis and anti-apoptotic function of growth factor receptors involves bbc3 as downstream target [61]. The data suggest that 532 nm laser generate sufficient heat in skin tissue to enable cells to act and repress bbc3 to adapt survival pathway by preventing apoptosis.

**Table 3:** Genes expressed following 532 nm laser exposure at low irradiance of 0.5 W/cm^2^.

Gene Symbol	Fold Regulation	p-Value	Predictive Pathway
Hes5	-15.3		notch pathway
Hey2	-2.19		
Heyl	-2.01		
Dab2	2.04	0.0322	wnt pathway
Mmp7	-11.9		
Bbc1	-11.4		p53 pathway
Egfr	-2.54		
Rb1	-2.29		
Fcer2	-5.45		jak/stat pathway
Ifng	-11.9		nfkb pathway
Tnf	-2.75		
Wnt1	-11.9		hedgehog pathway
Wnt2b	-4.86		
Wnt3a	-2.18	0.0273	
Epo	-11.89		hypoxia pathway
Car9	-7.17		
Olr1	-4.94		ppar pathway
Fabp1	-11.9		
Sorbs1	-2.05		

Notch signaling induces epidermal differentiation and required for hair follicle maintenance [62,63]. Hes 5 and hey2 are family of transcriptional repressors of notch pathway [63,64]. Hey2 and hes5 appear to play more important role in outer hair cells [65]. In the present study, hes5 and hey2 were found to be down regulated and the data suggest improper hair cell growth (Table 3 and Fig 5).

Skin wounds express wnt proteins during early phases of healing including wnt1, and wnt3 which remains present up to 7 days of injury in cutaneous wounds [66]. Further, Widelitz 2018 [67], reported that Wnt1 and Wnt3a favor with wnt/ β-catenin canonical pathways. In the present study, wnt1 and wnt3a were down regulated in skin tissue exposed to 532 nm laser and in contrast dab2 was upregulated (Table 3 and Fig 5). Higher expression of dab2 inhibits β-catenin signaling and cease wnt mediated proliferation thereby delaying wound healing [68]. On other hand, higher expression of wnt3a improves cutaneous repair. The observations obtained from this study suggest that lower expression of wnt3a with higher dab2 might result in delay in wound healing by negatively regulating canonical wnt pathway.

In canonical wnt/ β-catenin pathway, β-catenin is degraded in lack of wnt ligand in general but it is documented that in early phase of wound repair β-catenin can be activated by various growth factors such as TGFβ [53]. Stabilization of β-catenin after activation leads to its nuclear translocation binding to transcriptional co-activators LEF/TCF which further binds to promoter of target genes such as Cyclin D1, MT1-MMP, MMP7, Dkk1 [69]. Among these matrix metalloproteinases (MMPS) are essential for the wound closure and regulate TGFβ and TNF signaling for maintaining tissue remodeling, cleavage of extracellular proteins, activation of growth factors, migration of cells and neo-vascularization [70]. In early phase of wound healing, MMP7 helps in release of TNFα from macrophages to established local chemokine gradient [70]. In the present study, no change was observed in tnf gene expression and mmp7 was found to be down regulated significantly (Table 3 and Fig 5). The data suggest that mmp7 is essential for the activation of tnf following laser exposure and in absence of local chemokine gradient wound closure would be affected. Further, erythropoietin (epo) improves skin wound healing by activating TGFβ [71]. In this study, epo expression was under expressed which was concomitant with no change in TFGβ expression confirming delayed wound healing process at high energy laser exposure (Table 3 and Fig 5). The relationship between laser exposure and activation of TGF Beta pathway is well reported in patients and activation of TGFβ1 following laser treatment in patients is connected with wound healing process [72–75].

It is well documented that wnt and notch signaling pathways are critically involved in cutaneous repair and important for the regulation of migration, proliferation and differentiation of cells [76]. Further, activation of wnt/β-catenin and notch signaling has been observed in wound repair [77]. In one of the study, wnt/catenin and notch activation promoted wound closure and inhibition of either pathway lead into delayed wound closure [76].

Skin exposed to 532 nm green laser expressed wnt3a and dab2 differentially compared to control, low and moderate level of laser exposure. At higher level of laser exposure, it could be assumed that high heat energy may have been absorbed by the skin and expression of these genes is noteworthy. 532 nm laser induced repression of wnt3a (wnt/b-catenin activator) with higher dab2 expression (negative regulator of canonical Wnt/beta-catenin) suggest that at higher radiance there was shift from wnt canonical pathway to non-wnt canonical pathway with significant representation of genes from notch, nfkb, hedgehog, hypoxia, ppar and p53 pathways which might be due to high heat generated compared to moderate radiance exposure. Further studies are required to elucidate exact nature of contributions of these pathways in laser tissue interaction particularly 532 nm laser with skin tissue.

**Fig 6:**
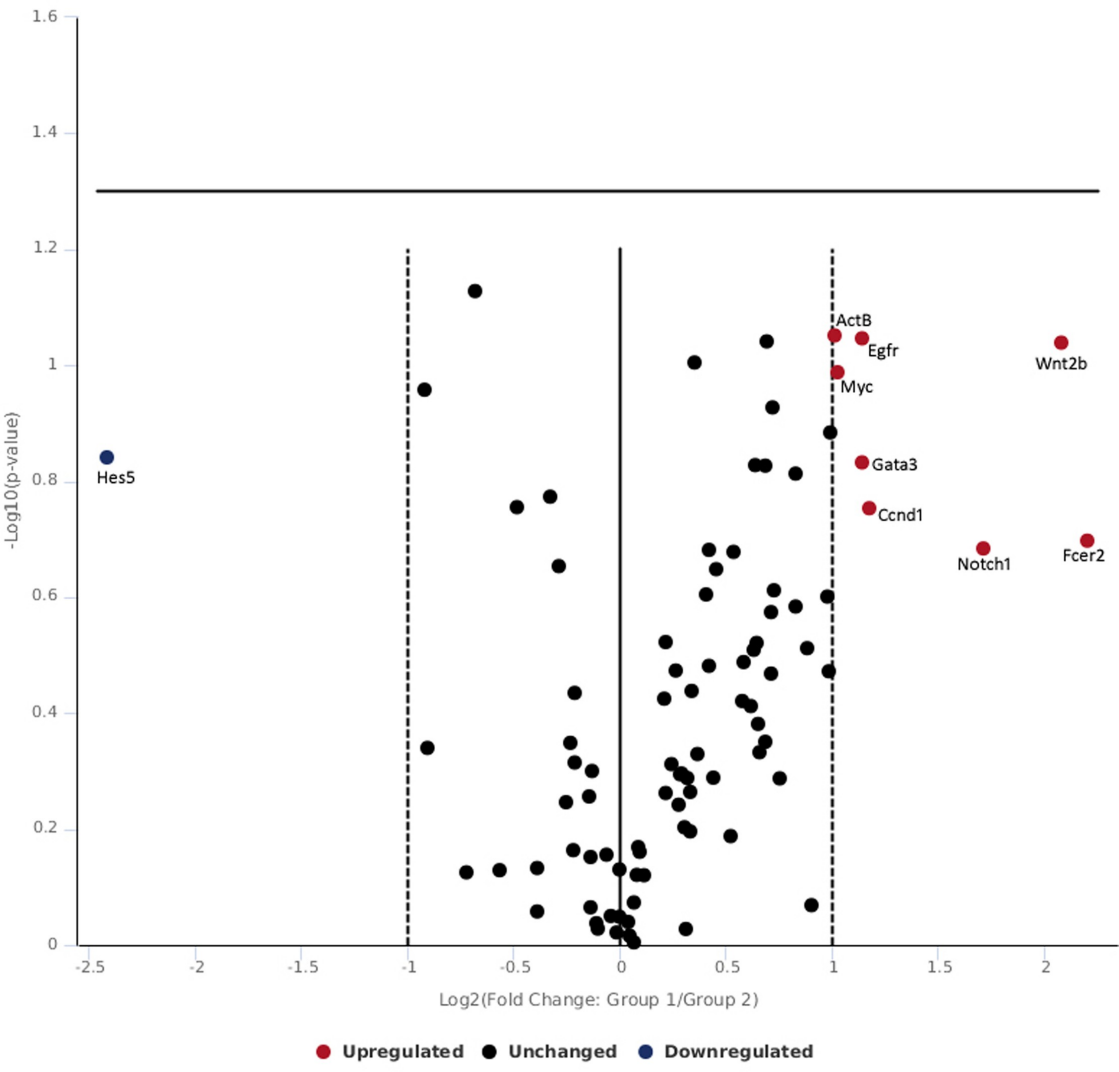
Volcano plot of intergroup comparison of genes expressed following 532 nm laser exposure at low irradiance (0.10 W/cm^2^) and moderate irradiance (0.25 W/cm^2^).

**Fig 7:**
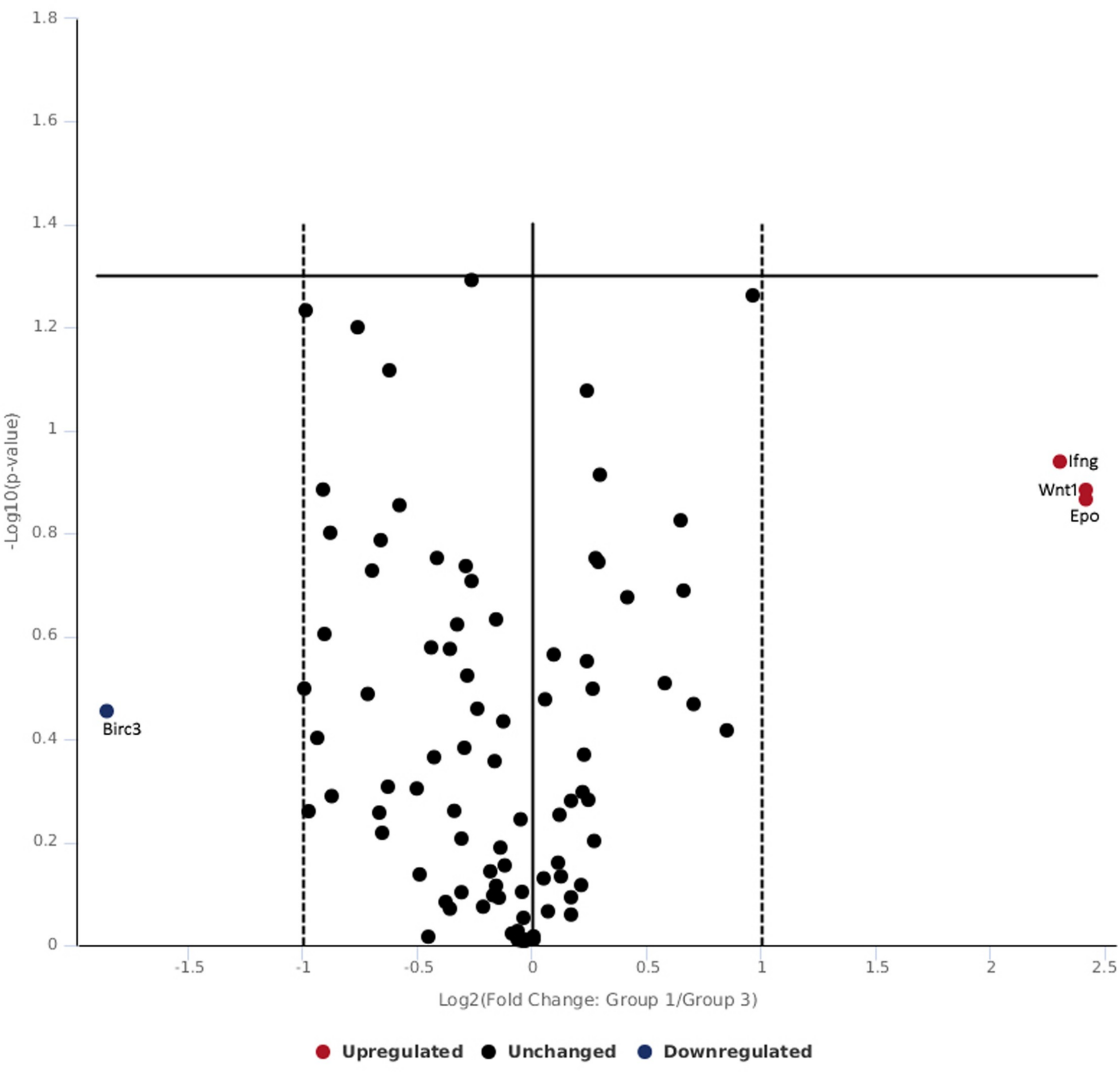
Volcano plot of intergroup comparison of genes expressed following 532 nm laser exposure at low irradiance (0.10 W/cm^2^) and high irradiance (0.50 W/cm^2^).

**Fig 8:**
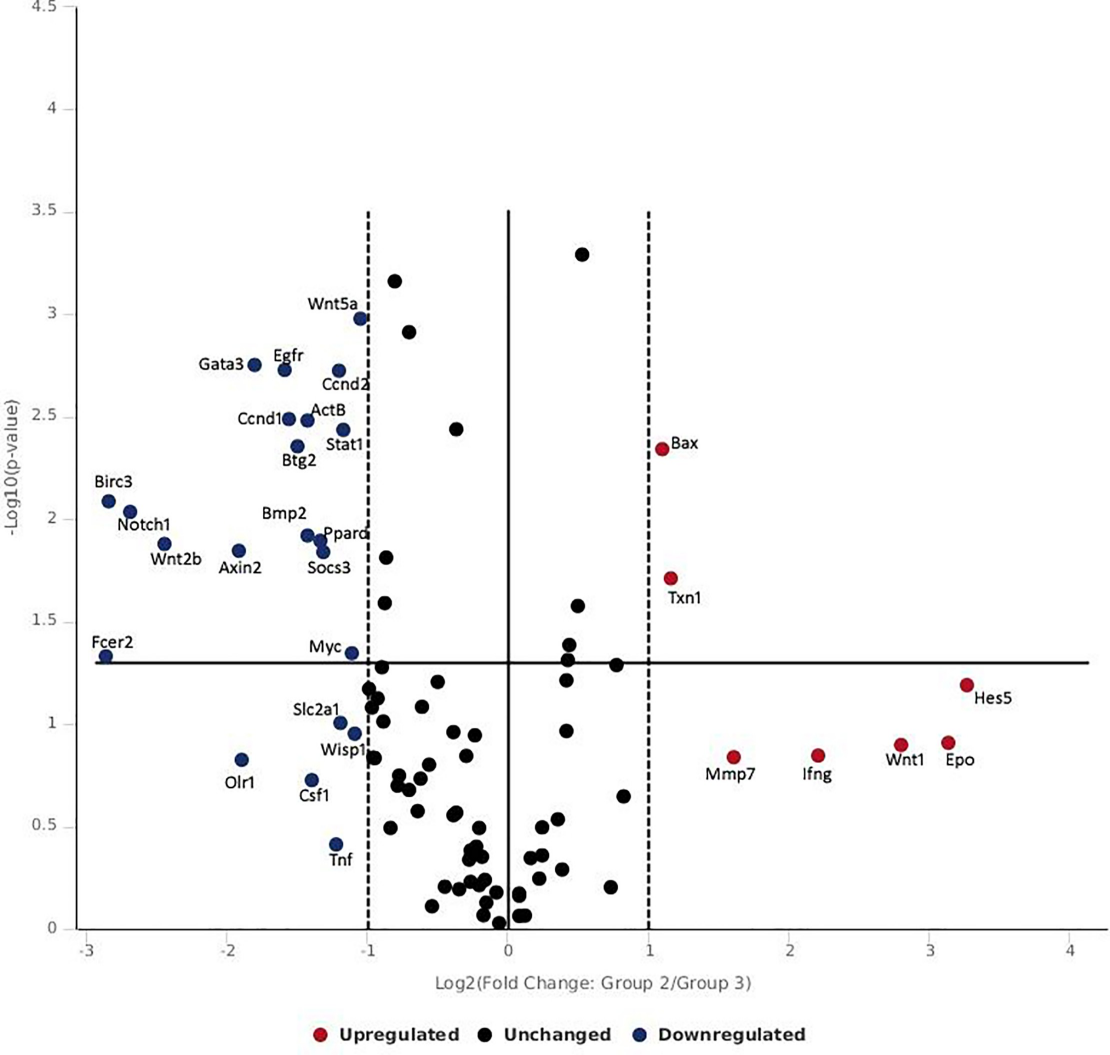
Volcano plot of intergroup comparison of genes expressed following 532 nm laser exposure at moderate irradiance (0.25 W/cm^2^) and high irradiance (0.50 W/cm^2^).

Inter-group data comparison of 0.1 W/cm^2^ (low radiance) and 0.25 W/cm^2^ (moderate radiance) indicate that wnt pathway (wnt2b) with notch pathway (notch1/egfr) were differentially expressed (Fig 6) however wnt pathway (wnt1/epo) was different without notch signaling when 0.1 W/cm^2^ (low radiance) was compared with 0.5 W/cm^2^ (high radiance group) (Fig 7). Further, comparison between moderate radiance group (0.25 W/cm^2^) and high radiance group (0.5 W/cm^2^) showed distinct expression of bax, txn1 and mmp genes (Fig 8). The findings indicate that impact on gene expression in rat skin was radiance dependent and there was shift in distinct and differential gene expression between low to moderate and high radiance. At low radiance classical skin repair and regeneration pathways expressed while at moderate radiance early wound healing genes (MMP7, wnt/notch, egfr) and genes of oxidative stress and anti-apoptosis pathways (txn1 and bax) were activated. However, at high radiance (0.5 W/cm^2^), ifn-gamma, wnt1 and epo were differentially expressed which are associated with wound healing, wound closure and vascular integrity.

**Fig 9:**
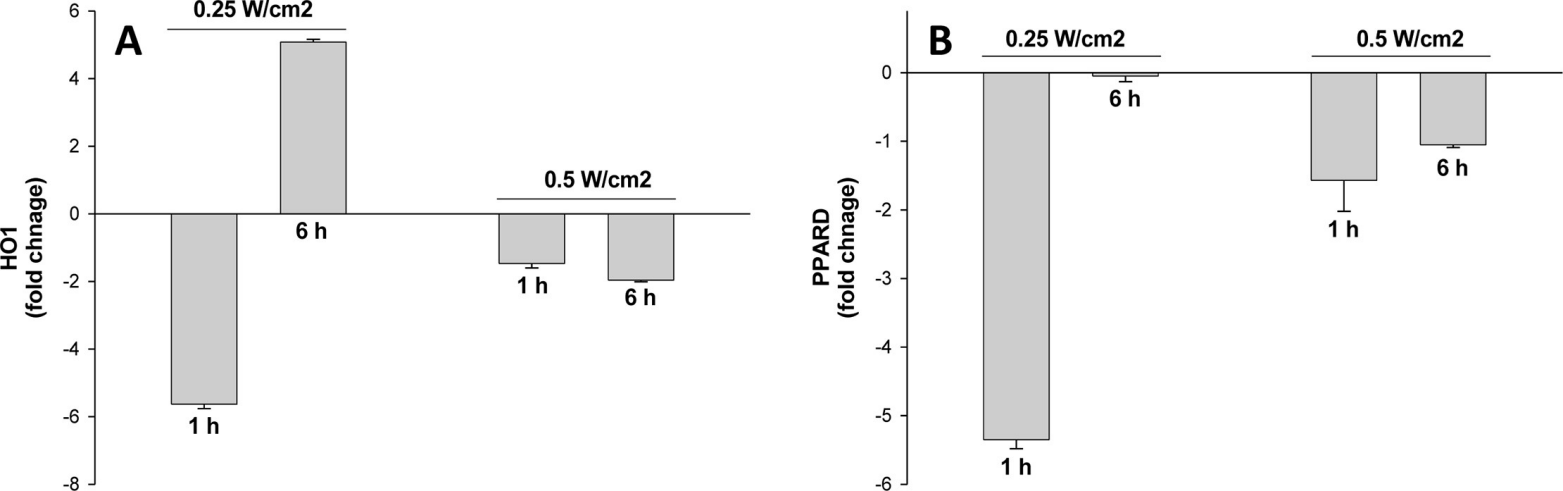
Validation of genes; ho1 gene (A) and ppard gene (B) using q-PCR in rat skin exposure 532 nm laser. The fold change for 0.1 W/cm^2^ exposure was negligible to unexposed group and hence not depicted on graph.

**Fig 10:**
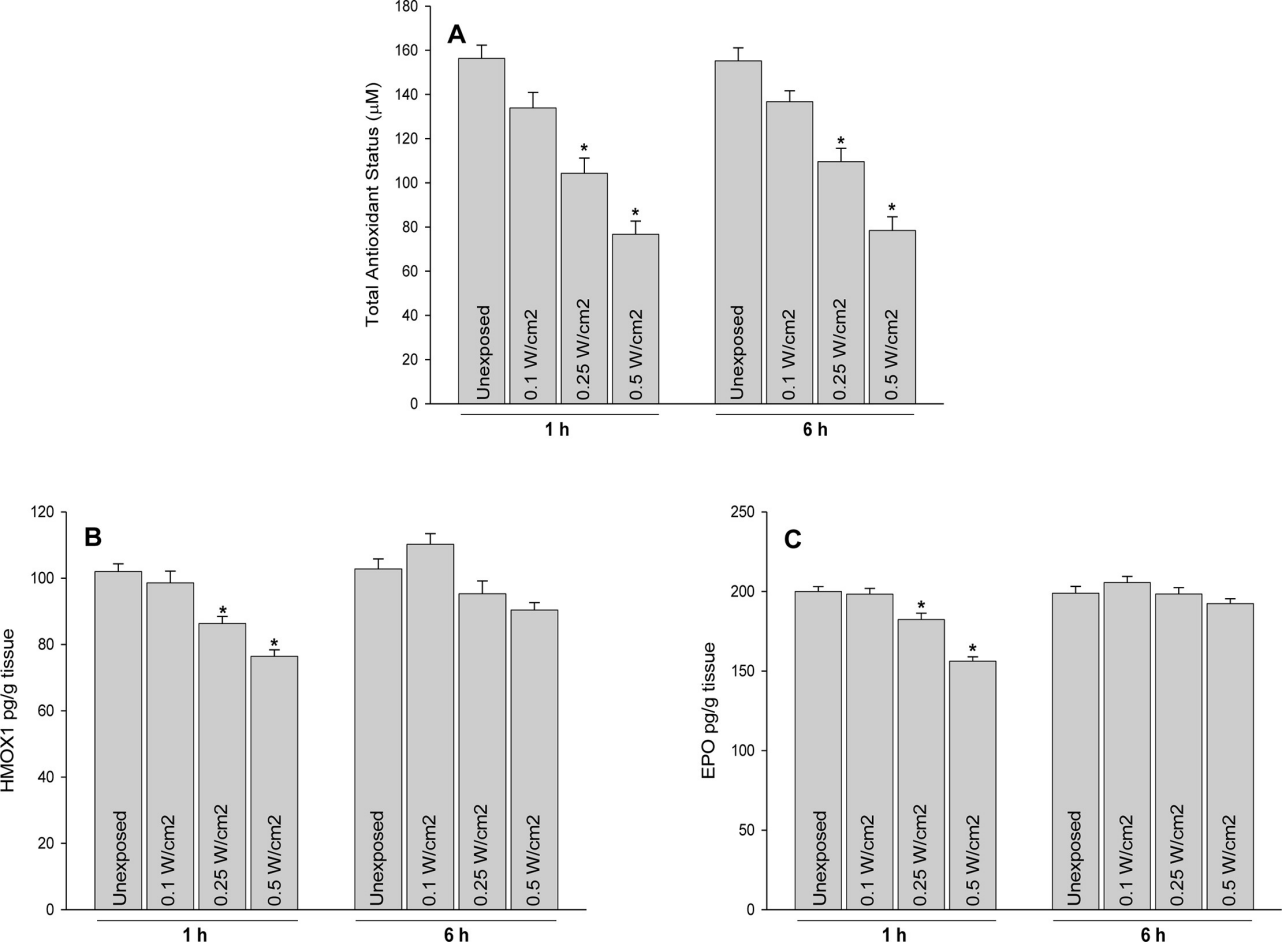
Effect of 532 nm laser exposure on total antioxidant status (A) and expression of hemeoxygenase 1 (B) and erythropoietin (C) in rat skin. *p<0.05.

The finding of gene expression studies were validated with two randomly picked genes pprad and ho1 using qPCR technique at radiances; 0.1 W/cm^2^, 0.25 W/cm^2^ and 0.5 Cm^2^ after 1 h and 6 h of laser exposure (Fig 9A and 9B) which authenticated data obtained from gene expression array studies. Further to corroborate findings obtained at gene expression, total antioxidant status and protein expressions of HO1 and EPO were studied following 532 nm laser exposure to rat skin. A decrease in total antioxidant levels were evident following 532 nm laser exposure which were radiance dependent (Fig 10A). Further, protein expression of heme oxygenase 1 (HO1) and erythropoietin (EPO) were measured for the substantiate effect of 532 nm laser at translation levels. The expression of HO1 and EPO were observed to be decreased in rat skin in radiance dependent manner after exposure to 532 nm laser (Fig 10B and 10C). The findings confirmed the impact of 532 nm laser was significant at 0.5 W/cm^2^ and corroborated findings obtained at genetic expression level. Further experimental studies are required to establish safety limits for handling of 532 nm laser devices for skin exposures and ethical use of military devices and other medical purposes.

## Conclusions

**Fig 11:**
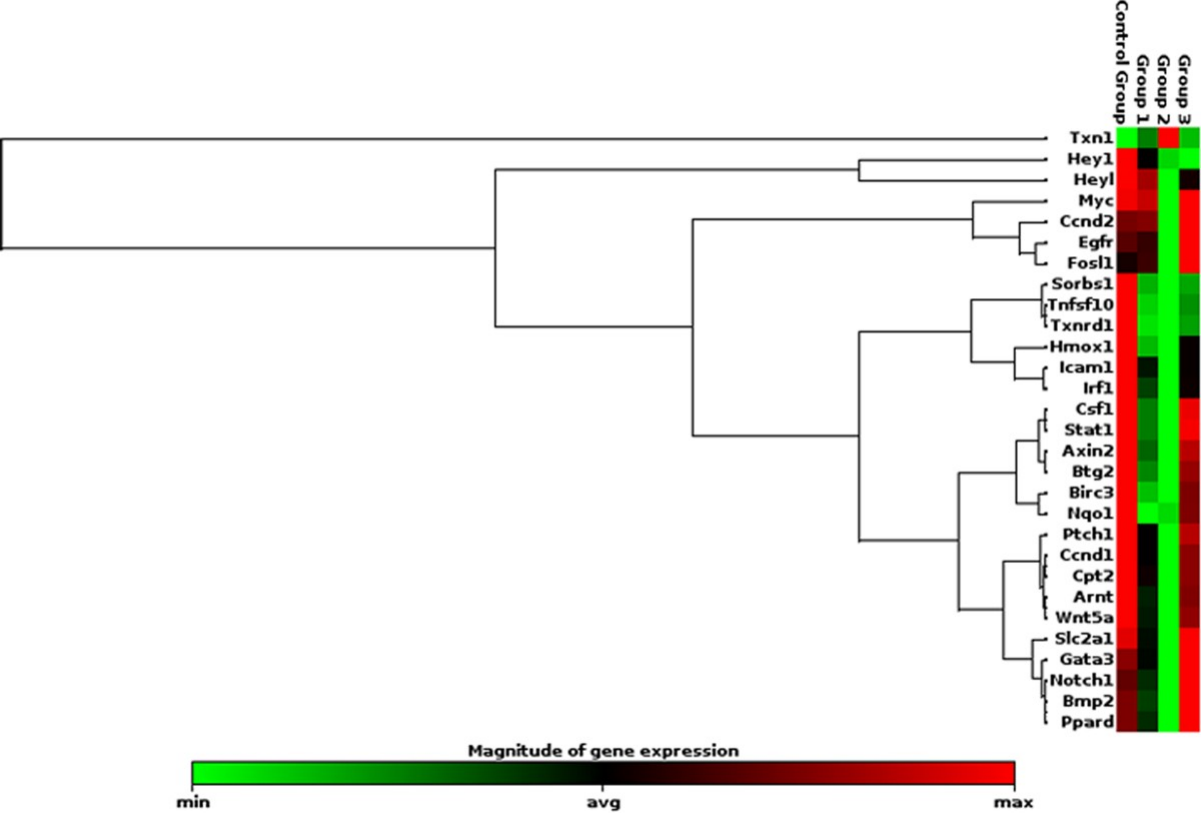
Clustergram of genes expressed significantly following 532 nm laser exposure at moderate irradiance of 0.25 W/cm^2^.

**Fig 12:**
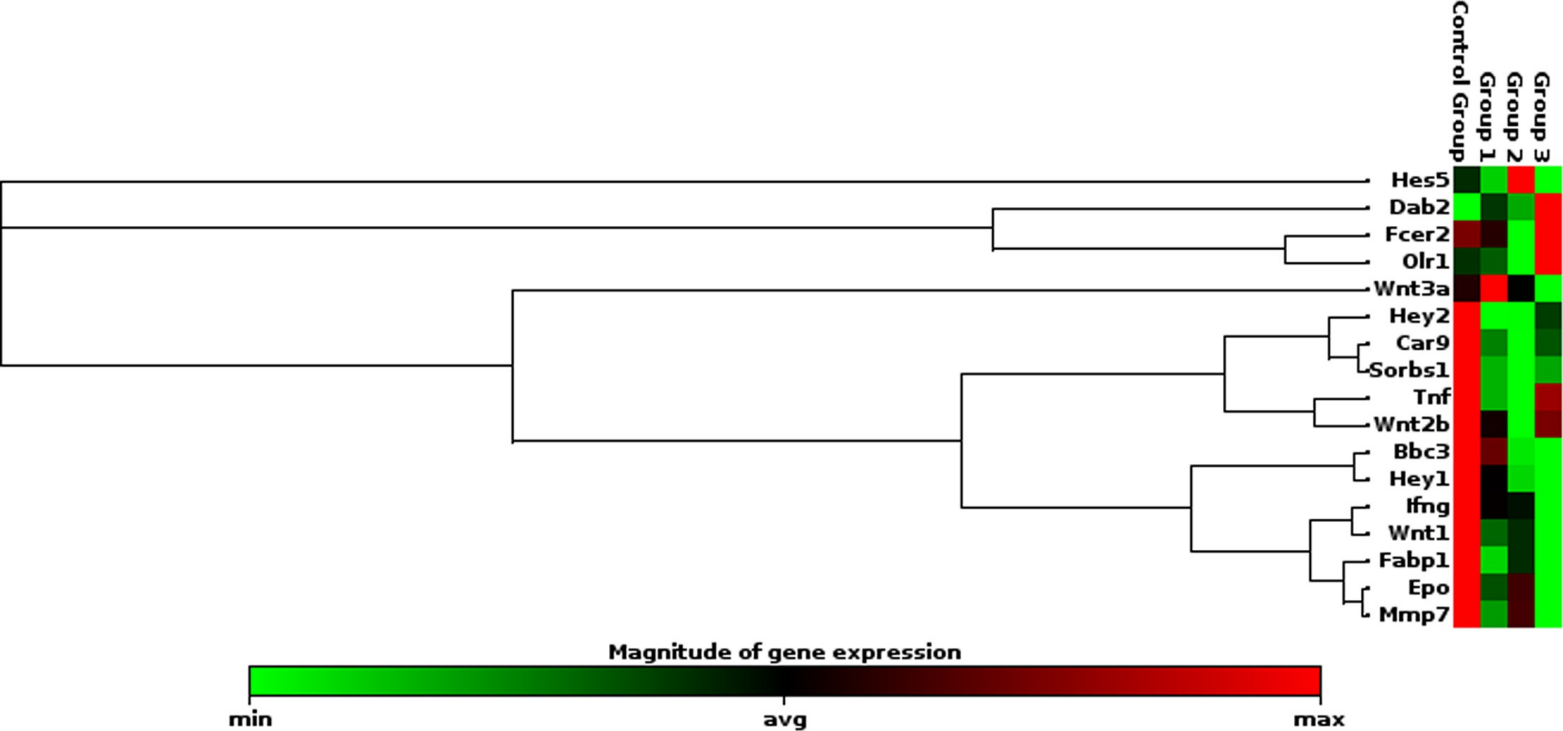
Clustergram of genes expressed significantly following 532 nm laser exposure at high irradiance of 0.50 W/cm^2^.

The rat skin exposed to laser beam showed distinct pattern of gene expression which was dependent on the radiance exposure. At the low level of 0.1 W/cm^2^ power densities, no change in gene expression was observed compared to unexposed skin. At moderate level, exposure of skin to 0.25 W/cm^2^, the genes related to cell survival, apoptosis, dermal inflammation; redox homeostasis and wound healing process were found to be regulated but the fold changes were not alarming (Fig 11). However, at 0.5 W/cm^2^ exposure level, significant repression of genes related to wound healing process (notch/wnt pathway) was recorded which may delay wound closure and healing (Fig 12). It was worth noting that there was shift from differential genes activated at moderate and high level of radiance exposure to skin which opens new avenue to configure pathway to counteract laser induced delay in wound healing and hair follicular damage.

The prospects of the study include: 1. The study address safety of 532 nm on rat skin and data would be useful to drawing safety limits for all prospective studies. 2. The study shows radiance dependent impact of 532 nm on rat skin and hence can be used as model test system for future biophysics studies. 3. The study opens new avenue to deal with laser-tissue interaction in wound healing and hair follicular damage and regeneration.
